# Associations of Composite Dietary Antioxidant Index and Dietary Inflammation Index with Cognitive Dysfunction in Older Chinese Adults: Results from China Health and Nutrition Survey in 2018

**DOI:** 10.3390/nu17213412

**Published:** 2025-10-30

**Authors:** Lina Huang, Zhihong Wang, Shuxia Yan, Qiuqin Wang, Liusen Wang, Ran Ye, Gangqiang Ding, Guihua Xu

**Affiliations:** 1 School of Nursing, Nanjing University of Chinese Medicine, Nanjing 210023, China; huangln@njucm.edu.cn (L.H.); nzyyanshuxia@163.com (S.Y.); qiuqin.wang@njucm.edu.cn (Q.W.);; 2 National Institute for Nutrition and Health, Chinese Center for Disease Control and Prevention, Beijing 100050, China; wangzh@ninh.chinacdc.cn (Z.W.); wangls@ninh.chinacdc.cn (L.W.)

**Keywords:** dietary quality, cognitive dysfunction, metabolic disorder, mediation effects

## Abstract

Background: Previous studies have shown that a diet with inflammatory and antioxidant properties can alter the risk of cognitive impairment. There are few studies using a large sample of the Chinese population. The specific relationship between inflammation, an antioxidant diet, and cognitive impairment remains unclear, and the potential impact of metabolic disorders remains to be determined. Methods: This is a cross-sectional study, with data from the China Health and Nutrition Survey (CHNS) in 2018. Individual and combined effects of the dietary inflammation index (DII) and composite dietary antioxidant index (CDAI) on cognitive impairment were assessed by binary logistic regression models. Nonlinear correlations and the inflection point were explored using restricted cubic splines (RCSs), and the mediation effects of triglyceride glucose–body mass index (TyG-BMI) were explored in greater depth using causal mediation analysis. Results: An increased CDAI was associated with a significantly decreased risk of cognitive impairment, at 0.68 (95%CI: 0.499–0.928). Contrary to this, the DII was positively associated with the risk of cognitive impairment, at 1.289 (95%CI: 1.03–1.613). The joint effects of the DII and CDAI indicated the minimal hazard effects on the risk of cognitive (0.787 (95%CI: 0.622–0.995)) impairment in subjects with low_DII + high_CDAI when compared with those with high_DII + low_CDAI. Furthermore, a significant nonlinear relationship was found between the CDAI and the risk of cognitive impairment, exhibiting an “L”-shaped curve (*p*-overall = 0.001, *p*-nonlinear = 0.007). However, no evidence was found for a nonlinear relationship between the DII and the risk of cognitive impairment. The mediation analysis did not reveal a mediating effect of TyG-BMI on the association between the CDAI and DII scores and the risk of cognitive impairment. Conclusions: Findings revealed that the CDAI could mitigate the adverse consequences of the DII on cognitive decline, which offers new insights into preventing early cognitive impairment through dietary intervention.

## 1. Introduction

The marked increase in the aging population has made Alzheimer’s disease (AD) an increasingly serious public health issue. It estimated that there was a substantial increase in the number of individuals diagnosed with dementia, from 57.4 million cases worldwide in 2019 to 152.8 million cases by 2050 [1]. In 2021, the estimated population living with dementia in China was 16.99 million individuals [2]. The early identification of cognitive dysfunction and subsequent early intervention have been shown to be conducive to the improvement of cognitive function and the prevention or delay of the occurrence and development of dementia; this is a key window period for the prevention and treatment of dementia [3,4]. Therefore, more research is needed to identify other potential risk factors.

Inflammation has been identified as a key mechanism in the development of cognitive dysfunction [5]. Whether it comprises dietary patterns, dietary composition, or compounds in the diet, diet seems to be related to the level of inflammation in the body [6,7]. The relationship between diet and inflammation in the body has been a subject of considerable interest [8,9]. Studies have demonstrated that a range of nutrients, including those with anti-inflammatory properties like selenium, vitamin D, and omega-3 fatty acids, and those with pro-inflammatory ones such as saturated fats and sodium are connected to cognitive health [6,10]. The dietary inflammatory index (DII) is a scientifically validated tool that evaluates dietary inflammation by combining the intakes of various nutrients that possess unique anti-inflammatory or pro-inflammatory qualities [11]. As indicated by the extant literature, higher DII levels have been demonstrated to be associated with an increased risk of both AD and mild cognitive impairment [12,13]. Furthermore, another primary cause of cognitive impairment is oxidative imbalance. The amount of oxidative stress can be altered by what we eat, which in turn can affect cognitive health. Various dietary antioxidants (e.g., vitamins C, E, and K and plant active substances) have been proven to have beneficial effects on cognitive abilities. The composite dietary antioxidant index (CDAI) is a validated composite score, derived from the cumulative intake of multiple dietary antioxidants, and it effectively mirrors the dietary antioxidant intake level [14]. Previous studies have shown a negative association between the CDAI and mild cognitive impairment (MCI) [15,16].

Evidence indicates a link between metabolic disorders and cognitive decline. Notably, the triglyceride–glucose (TyG) index offers a more precise evaluation of metabolic dysfunction compared to using glucose and triglyceride levels separately. The latest studies have shown that the TyG index is associated with MCI in type 2 diabetes, Parkinson’s disease, and acute stroke [17,18,19]. Moreover, the global prevalence of obesity has been identified as a contributing factor to cognitive decline in numerous studies. The TyG combined with body mass index (TyG-BMI) can enhance its predictive power for metabolic syndrome [20]. Therefore, we speculate that TyG-BMI may provide better evidence for identifying cognitive impairment. Recent research also indicates that there is an association between the TyG-BMI and AD pathology, cognition, and brain structures such as the hippocampus, suggesting that monitoring this index could be a crucial indicator for evaluating AD risk and offering timely interventions [21]. Therefore, we aim to investigate the interrelationships among the DII, CDAI, and cognitive impairment in a large Chinese population study. Additionally, considering the association between metabolic disorders and cognitive health, particularly the potential mediating role of metabolic dysfunction, this aspect remains unexplored.

In this research, we utilized large-scale population sample data in China to clarify the link between dietary quality (DII and CDAI) and cognitive impairment and subsequently examined the joint impact of co-exposure using the DII and CDAI. Furthermore, we investigated the mediating role of the metabolic function indicator TyG-BMI in the association between dietary quality and cognitive impairment.

## 2. Materials and Methods

### 2.1. Study Design and Population

Data for this research were derived from the China Health and Nutrition Survey (CHNS), which is an ongoing, long-term, large-scale open cohort study covering a total of sixteen provinces, autonomous regions, and municipalities. The first investigation took place in 1989 and was followed up in 1991, 1993, 1997, 2000, 2004, 2006, 2009, 2011, 2015, and 2018, respectively. More detailed information about the CHNS profile can be found in previous publications [22]. Approval for the study protocol was granted by the institutional review board of the National Institute for Nutrition and Health of Chinese Center for Disease Control and Prevention (ethics approval code 2018-004, 14 March 2018). Each participant signed an informed consent form. Professional cognitive function screening scales, including the Mini-Mental State Examination (MMSE), have only been used since 2018 to measure cognitive function. Therefore, in this study, we only selected the data from 2018 for relevant analysis. Participants aged 55 years and older with comprehensive data on socio-demographic characteristics, lifestyle, body measurements, dietary assessments, relevant blood indicators, and cognitive tests were included in the present study. Subjects with implausible energy intake levels by gender (>5000 kcal/day or <800 kcal/day for males; >4000 kcal/day or <600 kcal/day for females) were not included. The analysis was conducted on a final sample of 6019 subjects (5207 with normal cognitive function; 812 with cognitive impairment).

### 2.2. Dietary Inflammatory Index (DII)

An experienced investigator carried out an in-person interview utilizing three consecutive 24 h dietary recalls, along with food models, photographs, and other visual tools to assist participants in recording dietary intake information for each household member aged 2 years and older, including quantity consumed, cooking method, and dining location. The daily consumption of edible oils, sugar, salt, and sauces in the home was calculated by weighing the changes in the household food inventory. If the participant was too old or had cognitive issues that affected their ability to accurately record their 24 h dietary intake over the three days, their guardian or other relatives would meticulously record their daily dietary intake. The Chinese Food Composition Table was used to code and evaluate food recalls in order to determine the daily intake amounts for each food group and nutrients (included energy intake). Furthermore, depending on weighing records, the amount of edible oil and condiments consumed by each household member who ate meals at home during the survey was distributed depending on the family members’ energy intake percentage.

The DII is a scoring system that was created by Shivappa [11] and comprises 45 dietary parameters; however, the DII score can still be used even if there are fewer than 30 dietary parameters. Previous studies indicate that using 27 or 18 dietary parameters to assess the DII still allowed it to maintain its reliability [23,24]. Due to the types of dietary components examined in the CHNS being limited, 23 components were chosen for the DII calculation in the present study, including carbohydrates, proteins, fats, insoluble dietary fiber, cholesterol, saturated fatty acid, monounsaturated fatty acid, polyunsaturated fatty acid, vitamin A, vitamin C, vitamin E, thiamine, riboflavin, niacin, folic acid, calcium, phosphorus, potassium, magnesium, iron, zinc, selenium, β-carotene, anthocyanin, and soy isoflavone. The DII was calculated using the following steps [11,25,26]: (1) the nutrient residual model was used to calculate nutrient intake after energy adjustment; (2) Z score = (daily mean intake − global daily mean intake)/standard deviation; (3) the Z-score was converted to a percentile score (*p*-score) to reduce the influence of outliers and right-skewed effects; (4) Z score’ = *p*-score × 2 − 1; (5) a certain dietary component DII = (Z score’ × the inflammatory effect score of each dietary component); and (6) the total dietary DII value of an individual is equal to the sum of the DII values of each dietary component among the 23 dietary components. A higher DII value indicates a more pro-inflammatory diet; a lower value indicates a more anti-inflammatory diet.

### 2.3. Composite Dietary Antioxidant Index (CDAI)

The CDAI was assessed using the method developed by Wright [14]. This method covers six minerals and vitamins, including selenium, zinc, vitamins A, C, and E, and carotenoids in food. The CDAI was calculated using the following equation: CDAI = ((daily mean intake − global daily mean intake)/standard deviation). A higher CDAI value indicates stronger dietary antioxidants; a lower value indicates lower dietary antioxidants.

### 2.4. Assessment of Cognitive Function

The current study employed the Chinese version of the Mini-Mental State Examination (MMSE) to conduct cognitive assessments on all participants [27]. Previous studies have verified its applicability and effectiveness in the Chinese population. Trained investigators conducted the MMSE strictly in person, adhering to the guidelines and protocols, and participants completed it within 5–10 min. It is a 30-point test that is extensively used in clinical and research contexts to assess cognitive impairment. It includes basic tasks in several domains, such as language use and comprehension, basic motor skills, arithmetic calculations (such as serial subtractions of seven), time and place, and repeating lists of words. The classification criteria for cognitive impairment were as follows: illiteracy ≤ 17 points, primary school education ≤ 20 points, secondary school education (including secondary technical schools) ≤ 22 points, and university education (including junior colleges) ≤ 23 points.

### 2.5. Metabolic Dysfunction Indicators

The TyG-BMI is a reliable, sensitive, and specific substitute marker for insulin resistance, associated with fasting plasma glucose, serum triglyceride, and body mass index (BMI), determined as Ln [triglyceride (mg/dL)  × fasting plasma glucose (mg/dL)/2]  ×  BMI. BMI is calculated by weight(kg)/height(m)^2^ [28].

### 2.6. Assessment of Covariates

Researchers with specialized training employed questionnaires to collect information on age, gender, education level, physical activity, smoking status, and alcohol status over the past 12 months, as well as the residence area and community information. For data analysis, all these parameters were categorized. Gender categories: male and female; education level: below and primary school, middle school, high school and above; physical activities: occupational activities, household activities, leisure activities, and transportation activities. We classify the total amount of physical activity into low, medium, and high levels based on the tertile distribution. Smoking status and drinking status over the past year: yes and no; residence area: urban and rural. Additionally, twelve multidimensional components that represent the diversity of economic, social, demographic, and infrastructure features at the community level were used to calculate the community urbanicity index [29], which we divided into low, middle, and high levels based on the tertile distribution.

### 2.7. Statistical Analysis

Continuous variables with normal distribution were presented as mean  ±  standard deviation (SD) and used the z-test, while skewed distribution variables were expressed as medians (interquartile ranges) and used a non-parametric statistical hypothesis test including the Wilcoxon signed-rank test and the Kruskal–Wallis test. Categorical variables were expressed as n (%) and tested using the chi-squared test.

Binary logistic regression analyses were performed to calculate the odds ratios (ORs) and 95% confidence intervals (CIs) for CDAI and DII scores on cognitive impairment. Three models were created as follows: Model 1 was adjusted for sociodemographic covariates including age, gender, education level, residence area, and the community urbanicity index; Model 2 was further adjusted for statuses of current smoking and alcohol consumption, as well as the physical activity level; and Model 3 additionally adjusted for BMI and mean energy consumption. Note that in Model 3, which examines the relationship between the DII and cognitive impairment, energy adjustment has already been accounted for in the calculation of the DII. Consequently, only BMI is further adjusted in Model 3. To evaluate the combined impact of the CDAI and DII on cognitive impairment, the CDAI and DII were individually divided into two groups by a cut-off value of 0 based on an index calculation, where the scores of 0 indicated that the intake of each dietary component matched the global average. To evaluate the combined effects between the DII and CDAI on cognitive impairment, based on an index calculation, the DII and CDAI were individually categorized into two groups with a cut-off value of 0, where the scores of 0 represent the intake of each dietary component being equivalent to the global per capita level. Subsequently, pairwise combinations were executed, which resulted in the establishment of the four following subgroups: high_DII + low_CDAI, high_DII + high_CDAI, low_DII + low_CDAI, and low_DII + high_CDAI. The joint effects of co-exposure using the DII and CDAI in the latter three subgroups were evaluated using the first subgroup (high_DII + low_CDAI) as the reference, with adjustments made for covariates as specified in Model 3, which examines the relationship between the DII and cognitive impairment as above.

A restricted cubic spline function was utilized to explore the potential nonlinear association between the CDAI and DII score and cognitive impairment, with adjustments made for the same covariates as in the preceding analysis.

Finally, to explore whether TyG-BMI plays a mediating role between the CDAI, DII and cognitive impairment, we adopted the simple mediation model. In this model, X can influence Y through two distinct pathways. These pathways are identified by tracing the routes from X to Y, ensuring that the direction of travel aligns with the direction indicated by the arrows. The first pathway, termed the direct effect, connects X to Y without involving M. The second pathway, known as the indirect effect, involves X influencing Y through an intermediary variable, M. This indirect effect illustrates how X affects Y via a causal sequence, whereby X impacts M, which subsequently affects Y. The regression coefficients a, b, and c′ are assigned to the variable X within the model to estimate the outcomes; c′ quantifies the direct effect of X on Y. The indirect effect of X on Y, mediated through M, is represented by the product of a and b. The total effect of X denoted as c = ab + c′ [30].

SAS software (version 9.4, SAS Institute Incorporated, Cary, NC, USA) was utilized for all statistical analyses. For the mediation and moderation model, IBM SPSS Statistics (version 21, IBM Corp., Armonk, NY, USA) and the PROCESS plug-in by Hayes’ team were employed, opting for Model 4 for the mediation analysis. A *p*-value of <0.05 was considered to be statistically significant.

## 3. Results

### 3.1. Characteristics of Study Population by Cognitive State

A total of 6019 subjects aged 55 years and older were included in the present study, which included 812 (13.5%) cases of cognitive impairment. The prevalence of cognitive impairment exhibited significant variation across gender, age, residence area, community urbanicity index, educational level, physical activity, smoking and alcohol status, BMI, and total energy intake (all *p* < 0.05). In addition, there were significant differences in the prevalence of cognitive impairment among the quartiles of the CDAI and DII scores (*p* < 0.05) (Table 1).

### 3.2. Associations Between CDAI, DII, and Risk of Cognitive Impairment

The associations between the CDAI, DII, and risk of cognitive impairment are showed in Table 2. The CDAI and DII were divided into quartiles in the analysis. Compared with the first quartile, the risk of cognitive impairment reduces in the fourth quartile of the CDAI [OR (95%CI) = 0.68 (0.499, 0.928)], while the risk of cognitive impairment had a strong positive association with the fourth quartile of the DII [OR (95%CI) = 1.289 (1.03, 1.613)].

### 3.3. Joint Effects of DII and CDAI on Cognitive Foundation

Due to the substantial but contrary effects of the DII and CDAI on cognitive function, their combined impact was subsequently analyzed using pairwise combinations. There was a notable reduction in the risk of cognitive impairment [OR (95%CI) = 0.787 (0.622, 0.995)] among subjects in the low_DII + high_CDAI group compared to those in the high_DII + low_CDAI reference group after considering the impact of confounding factors (gender, age, residence area, community urbanicity index, education level, physical activity, smoking and alcohol status, BMI) (Table 3).

### 3.4. Dose–Response Relationship Between CDAI, DII, and Prevalence of Cognitive Impairment

**Figure 1:**
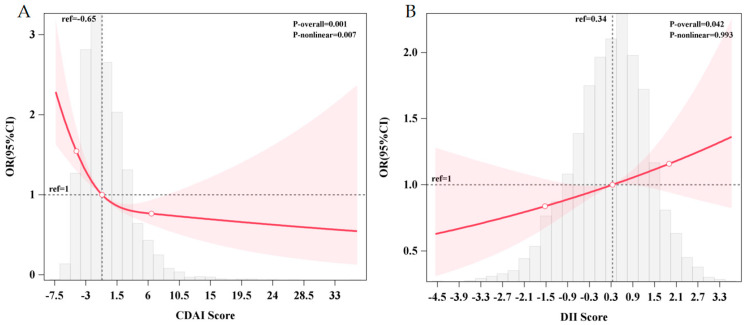
The dose–response relationship between the CDAI (**A**), DII (**B**), and the prevalence of cognitive impairment. (**A**) Restricted cubic spline using logistic regression adjusted for age, gender, education level, residence area, community urbanicity index, smoking and alcohol status, physical activity level, BMI, and mean energy intake. (**B**) Restricted cubic spline using logistic regression adjusted for age, gender, education level, residence area, community urbanicity index, smoking and alcohol status, physical activity level, and BMI. (The gray area represents the distribution density of CDAI scores in the study sample, the red line represents the nonlinear trend of the RCS-fitted OR values as a function of CDAI scores, the red area represents the 95% confidence interval for the RCS estimate).

Following covariate adjustment, there is a significant nonlinear relationship between the CDAI and the risk of cognitive impairment in RCS (*p*-overall = 0.001; *p*-nonlinear = 0.007), and it presents as “L”-shaped nonlinear. As the CDAI score increased, the risk of cognitive impairment declined. When the threshold of the CDAI was less than −0.65, the risk of cognitive impairment was increased, while when it was more than −0.65, the risk of cognitive impairment was reduced (Figure 1A). However, there seems to be no evident nonlinear association between the DII score and the risk of cognitive impairment (*p*-overall = 0.042; *p*-nonlinear = 0.993).

### 3.5. Mediation Effects of TyG-BMI on CDAI– and DII–Cognitive Function Associations

**Figure 2:**
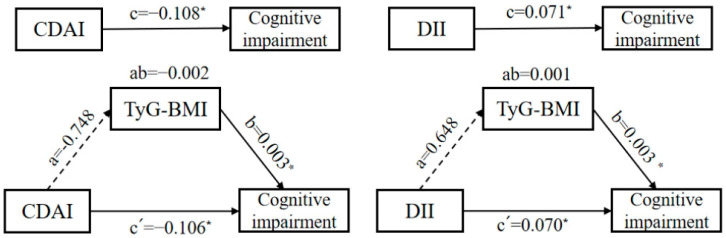
A diagram of the simple mediation models between the CDAI, DII, and cognitive impairment among Chinese adults. (The mediator is TyG-BMI. The direction is indicated by arrows. A solid line represents a significant coefficient and a confirmed pathway, while a dashed line represents an insignificant coefficient and an unconfirmed pathway. Omit the covariables and error terms. * *p* < 0.05.)

Based on the simple mediating effect model with covariates (age, gender, education level, residence area, community urbanicity index, smoking and alcohol status, physical activity level), the pathway “CDAI/DII → TyG-BMI → cognitive impairment” was preliminarily explored. Figure 2 demonstrates that the total effect coefficient between the CDAI and cognitive impairment was −0.108, and the direct effect and the indirect effect coefficients were −0.106 and −0.002, respectively. The total effect and the direct effect were statistically significant (*p* < 0.05); the indirect effect was not statistically significant (*p* > 0.05). The total effect coefficient between the DII and cognitive impairment was 0.071, and the direct effect and the indirect effect coefficients were 0.070 and 0.001, respectively. The total effect and the direct effect were statistically significant (*p* < 0.05), and the indirect effect was not statistically significant (*p* > 0.05). Therefore, the mediating effects of TyG-BMI in both pathways were not established.

## 4. Discussion

In our study, we found that the CDAI was associated with a significantly decreased risk of cognitive impairment. And this relationship remained even after adjusting for other covariates, indicating that the CDAI is a protective factor for cognitive function. In addition to this, this study found when evaluating the correlation between the CDAI and cognitive impairment, a dose–response relationship was discovered, which showed an “L”-shaped nonlinear negative correlation, and the inflection point was −0.65. Furthermore, we also concluded that the DII increases the risk of cognitive impairment. We did not see the anticipated dose–response effect and nonlinear relationship between the DII score and the presence of cognitive impairment.

As an indicator reflecting the overall antioxidant potential of dietary nutrients, research on the relationship between the CDAI and cognitive impairment is still relatively rare, having been mainly conducted on the American population. All these studies have shown that the CDAI can mitigate age-related cognitive decline, supporting cognitive health in the elderly [16,31,32]. Other research also showed that a higher dietary CDAI was associated with lower odds of cognitive impairment in later life in a Chinese population in Singapore [33]. These conclusions are in perfect agreement with the results of the Chinese population in our study. In addition, intervention studies have also found that an antioxidant-rich diet, compound nutritional supplements, and single nutrients can improve cognitive functions. In addition, we also found a dose–response-negative relationship between the CDAI and cognitive impairment. The foods emphasized in the MIND diet are rich in specific compounds that combat oxidative stress. These compounds not only neutralize free radicals but also enhance cellular signaling pathways that promote neuroplasticity [34]. There have been multiple studies that have shown an association between the MIND diet and a decreased risk of cognitive decline. A three-year clinical trial showed there was a significant improvement in cognition in the MIND diet group during the first two years of the study, suggesting that the diet may continue to improve cognition or delay cognitive decline [35]. MIND diet intervention can reverse the destructive effects of obesity on cognition and brain structure [36]. Evidence suggested that antioxidant-rich compound nutritional supplements can also improve cognitive function. Vitamin C combining tea polyphenols effectively improved the cognitive function of patients [37]. Vitamin E combined with lycopene significantly improved the cognitive function of MCI patients [38]. There are also inconsistent research conclusions. A six-year follow-up on the Prevention of Alzheimer’s Disease by Vitamin E and Selenium trial showed that there was no evidence that antioxidant supplements of vitamin E or selenium, either alone or in combination, prevented dementia [39].

There have been minimal efforts to study the connection between DII scores and cognitive impairment in large, representative populations to date. A large-sample (based on 7085 individuals) study from the Women’s Health Initiative Memory Study showed that higher DII scores were linked to more significant cognitive decline and an increased risk of developing cognitive impairment [40]. However, it focused on the female population and lacked representativeness of the population. UK Biobank (n = 166,377) showed that a higher DII was associated with a higher risk of all-cause dementia and AD [41]. Previous related studies (including from China) have employed relatively small samples, typically involving several hundred participants; however, they reached similar conclusions [42,43,44]. Consistent with previous studies, our data demonstrate that the DII had a positive association with the risk of cognitive impairment.

The mechanisms by which diets influence cognition may be indirect. Recent research has found that higher levels of TyG-BMI are associated with a lower risk of AD [21]. Therefore, we suspect that TyG-BMI may play a mediating role between diet and cognitive impairment. But our results show that no mediating effect of TyG-BMI was found either between the CDAI and cognitive impairment or between the DII and cognitive impairment. It may be because the CDAI and DII do not influence cognition through the TyG-BMI pathway. Current evidence indicates that diet can exert a bidirectional regulatory effect on cognition through oxidative stress and chronic inflammation via two core pathways. Reduced dietary inflammatory potential may also directly decrease chronic inflammation associated with neurodegenerative diseases such as Alzheimer’s disease [45,46]. In the WHI cohort, higher DII scores were associated with elevated concentrations of inflammatory markers, including IL-6, TNF-α, and hs-CRP, which can cross the blood–brain barrier, induce neuroinflammation, and damage neurons and synaptic function, ultimately leading to cognitive decline [40,47,48]. The active components in antioxidant foods (such as vitamins C and E, polyphenols, and carotenoids) can scavenge free radicals to reduce oxidative damage to neurons caused by reactive oxygen species, protect mitochondrial function, and maintain energy metabolism in nerve cells [10,49]. Antioxidant-rich foods also have significant anti-inflammatory effects, inhibiting the expression of pro-inflammatory factors such as IL-6, TNF-α, and NF-κB; regulating gut microbiota; reducing intestinal permeability; and lowering systemic inflammation [50,51]. The aforementioned mechanism may provide molecular evidence for the failure of TyG-BMI to mediate the effects of the CDAI and DII on cognitive changes in this study. However, a strong causal inference cannot be made from the conclusion of a single study.

Moreover, this study has several limitations. Firstly, although this study utilized a large sample of older adult participants from several regions in China, enhancing its statistical power and yielding robust results, it remains a cross-sectional study where bias in the findings is unavoidable. Meanwhile, mediation analysis has limitations regarding causal directionality, particularly in cross-sectional studies, which cannot establish the temporal sequence of a causal chain linking dietary quality (CDAI, DII), TyG-BMI, and cognitive impairment. Cross-sectional mediation analysis can only indicate a statistical association pathway and cannot support causal mediation. Large-scale longitudinal research is necessary to further explore how the CDAI and DII influence cognitive health and shed light on the black box. Secondly, for the older adults, three consecutive 24 h dietary assessments are prone to recall bias and may involve measurement errors and inaccuracies. Thirdly, although we adjusted for confounding factors, we still lack data on factors such as social activities that may influence cognitive health. Future research should account for these important confounding factors to more comprehensively elucidate the relationship between dietary intake and cognitive impairment.

## 5. Conclusions

In conclusion, a healthy diet is crucial for preventing and treating cognitive impairment. Our findings suggest that adherence to a high-CDAI diet is associated with a lower risk of cognitive impairment in an L-shaped nonlinear relationship, while a high-DII diet is associated with greater severity. These findings underscore the necessity of dietary strategies that limit pro-inflammatory components while ensuring adequate intake of antioxidant-rich components. Future efforts should focus on conducting additional randomized controlled trials or large-scale cohort studies to confirm this finding, which is essential for developing precise and effective strategies for preventing and treating cognitive impairment, ultimately improving the quality of life and health outcomes for individuals with cognitive impairment.

## Figures and Tables

**Table 1:** Characteristics of study population by cognitive state.

Factors		Total (N = 6019)	Cognitive State		*p*
			Controls (N = 5207)	Cases (N = 812)	
Gender, n (%)					0.0008
	Male	3229 (53.65)	2749 (52.79)	480 (59.11)	
	Female	2790 (46.35)	2458 (47.21)	332 (40.89)	
Age (years), Mean ± SD		65.9 ± 7.6	65.2 ± 7.2	69.9 ± 8.8	<0.0001
Residence area, n (%)					<0.0001
	Urban	3680 (61.14)	3113 (59.78)	567 (69.83)	
	Rural	2339 (38.86)	2094 (40.22)	245 (30.17)	
Urbanicity index, n (%)					<0.0001
	Low	2008 (33.36)	1664 (31.96)	344 (42.36)	
	Middle	1998 (33.19)	1729 (33.21)	269 (33.13)	
	High	2013 (33.44)	1814 (34.84)	199 (24.51)	
Educational level, n (%)					<0.0001
	~Primary school	2692 (44.73)	2267 (43.54)	425 (52.34)	
	Middle school	1713 (28.46)	1449 (27.83)	264 (32.51)	
	High school~	1614 (26.82)	1491 (28.63)	123 (15.15)	
Physical activity, n (%)					<0.0001
	Low	2002 (33.27)	1630 (31.3)	372 (45.87)	
	Middle	2010 (33.40)	1801 (34.59)	209 (25.77)	
	High	2006 (33.33)	1776 (34.11)	230 (28.36)	
Smoking status, n (%)					0.0114
	No	4545 (75.51)	3903 (74.96)	642 (79.06)	
	Yes	1474 (24.49)	1304 (25.04)	170 (20.94)	
Drinking status, n (%)					<0.0001
	No	4603 (76.47)	3937 (75.61)	666 (82.02)	
	Yes	1416 (23.53)	1270 (24.39)	146 (17.98)	
BMI (kg/m^2^)		24.4 (22.0, 26.8)	24.5 (22.2, 26.9)	23.6 (21.0, 26.4)	<0.0001
Energy intake (kcal/day)		1846.42 (1471.32, 2311.92)	1859.98 (1493.34, 2328.44)	1748.76 (1369.26, 2173.58)	<0.0001
CDAI, n (%)					<0.0001
	Q1	1504 (24.99)	1242 (23.85)	262 (32.27)	
	Q2	1505 (25.00)	1287 (24.72)	218 (26.85)	
	Q3	1505 (25.00)	1312 (25.2)	193 (23.77)	
	Q4	1505 (25.00)	1366 (26.23)	139 (17.12)	
DII, n (%)					0.0303
	Q1	1504 (24.99)	1333 (25.6)	171 (21.06)	
	Q2	1505 (25.00)	1300 (24.97)	205 (25.25)	
	Q3	1505 (25.00)	1294 (24.85)	211 (25.99)	
	Q4	1505 (25.00)	1280 (24.58)	225 (27.71)	
TyG-BMI, median (Q1, Q3)		215.77 (189.88, 242.90)	208.73 (179.67, 238.39)	216.9 (191.404, 243.39)	

Abbreviations: Q = quartile. Data of categorical variables are expressed as number (%); mean ± SE and median (interquartile ranges) are used for skewed distribution variables.

**Table 2:** Associations of dietary quality index (CDAI and DII) with cognitive state.

Dietary Quality Index		Model 1	Model 2	Model 3
		OR (95% CI)	OR (95% CI)	OR (95% CI)
CDAI	Q1	Ref	Ref	Ref
	Q2	0.978 (0.797, 1.201)	1.006 (0.818, 1.238)	0.995 (0.800, 1.237)
	Q3	0.881 (0.713, 1.088)	0.920 (0.743, 1.14)	0.901 (0.703, 1.155)
	Q4	0.671 (0.533, 0.846)	0.704 (0.558, 0.889)	0.680 (0.499, 0.928)
DII	Q1	Ref	Ref	Ref
	Q2	1.199 (0.958, 1.501)	1.184 (0.945, 1.484)	1.167 (0.931, 1.463)
	Q3	1.208 (0.965, 1.511)	1.178 (0.940, 1.476)	1.165 (0.930, 1.460)
	Q4	1.300 (1.042, 1.622)	1.259 (1.008, 1.574)	1.289 (1.030, 1.613)

Abbreviations: Q = quartile. Model 1: adjusted for age, gender, education level, residence area, and community urbanicity index; Model 2: based on Model 1, further adjusted for smoking status and alcohol status, as well as physical activity level; Model 3 of CDAI: based on Model 2, adjusted for BMI and mean energy intake. Model 3 of DII: based on Model 2, adjusted for BMI.

**Table 3:** Joint effects of DII and CDAI on cognitive foundation.

Dietary Quality Index		Model 1	Model 2	Model 3
		OR (95% CI)	OR (95% CI)	OR (95% CI)
CDAI + DII	high_DII + low_CDAI	Ref	Ref	Ref
	high_DII + high_CDAI	0.867 (0.697, 1.078)	0.892 (0.716,1.110)	0.966 (0.740, 1.262)
	low_DII + low_CDAI	0.921 (0.740, 1.146)	0.930 (0.746,1.158)	0.915 (0.733, 1.143)
	low_DII + high_CDAI	0.720 (0.580, 0.895)	0.752 (0.604, 0.935)	0.787 (0.622, 0.995)

## Data Availability

The article contains the original contributions from the study; for more information, please contact the corresponding author.

## Abbreviations

The following abbreviations are used in this manuscript:

CHNS	the China Health and Nutrition Survey
CDAI	composite dietary antioxidant index
DII	dietary inflammation index
RCS	restricted cubic splines
TyG	triglyceride–glucose
BMI	body mass index
TyG-BMI	triglyceride glucose–body mass index
AD	Alzheimer’s disease
MMSE	the Mini-Mental State Examination
SD	standard deviation
